# Infection of Human Coronary Artery Endothelial Cells by Group B Streptococcus Contributes to Dysregulation of Apoptosis, Hemostasis, and Innate Immune Responses

**DOI:** 10.1155/2011/971502

**Published:** 2011-02-06

**Authors:** Claudia Beyrich, Jürgen Löffler, Anna Kobsar, Christian P. Speer, Susanne Kneitz, Martin Eigenthaler

**Affiliations:** ^1^Institute of Clinical Biochemistry and Pathobiochemistry, University of Wuerzburg, D-97080 Wuerzburg, Germany; ^2^Medical Hospital II, University of Wuerzburg, D-97080 Wuerzburg, Germany; ^3^University Children's Hospital, University of Wuerzburg, D-97080 Wuerzburg, Germany; ^4^Laboratory for Microarray Applications, University of Wuerzburg, D-97080 Wuerzburg, Germany

## Abstract

Early onset sepsis due to group B streptococcus leads to neonatal morbidity, increased mortality, and long-term neurological deficencies. Interaction between septicemic GBS and confluent monolayers of human coronary artery endothelial cells (HCAECs) was analyzed by genome wide expression profiling. In total, 124 genes were differentially expressed (89 upregulated, 35 downregulated) based on a more than 3-fold difference to control HCAEC. Regulated genes are involved in apoptosis, hemostasis, oxidative stress response, infection, and inflammation. Regulation of selected genes and proteins identified in the gene array analysis was confirmed by Real-time RT-PCR assay (granulocyte chemotactic protein 2), ELISA (urokinase, cyclooxygenase 2, granulocyte chemotactic protein 1), and western blotting (Heme oxygenase1, BCL2 interacting protein) at various time points between 4 and 24 hours. These results indicate that GBS infection might influence signalling pathways leading to impaired function of the innate immune system and hemorrhagic and inflammatory complications during GBS sepsis.

## 1. Introduction

Pneumonia, sepsis, meningitis due to infection with GBS are common causes of morbidity, mortality, and long-term neurological sequelae in neonates [1]. Vaginal smears of up to 40% of pregnant women show colonization with *S. agalactiae, *50 to 70% of their children will be colonized postpartum [2, 3]; up to 2 newborns per 1000 live births will develop GBS infection [4, 5], which is characterized by unspecific clinical signs such as temperature instability, respiratory distress, palor, and abdominal symptoms. The majority of neonates develop early onset sepsis within the first 24 hours of life, which rapidly progresses in septic shock and hemorrhage. Depending on the gestational age, case fatality rate can increase up to 30 percent in preterm infants <32 weeks of gestation [6, 7]. More than 50% of affected newborns suffer from neurological deficencies like sight or hearing loss and mental retardation. In addition 19,000 cases of GBS disease occur annually in the United States, and up to 35% of the symptomatic infections in elderly and immunosuppressed patients suffer from chronic diseases [8]. 

GBS has characteristic virulence factors, including the ability to bind to extracellular matrix components, such as fibronectin, fibrinogen, and laminin [9], allowing to pass cellular barriers and to become invasive. 

Following bacteremia, immune response is partially insufficient, presumably due to immature innate immunity of the neonate [10]. Lipopolysaccharides of Gram negative bacteria are able to modify the barrier function of endothelial cells [11]. Lembo et al. could previously demonstrate that GBS are able to penetrate the blood-brain barrier by targeting human brain microvascular endothelial cells [12]. However it is unclear if GBS is also able to invade human coronary artery endothelial cells. Endothelial dysfunction is a major component in the pathophysiology of septicemic GBS infection [13, 14]. Therefore, analysis of the interaction between GBS and endothelial cells is of major interest, especially the septic and haemorrhagic complications in newborns. Genome wide expression profiling allows detailed insights in the pathogenesis of GBS sepsis and helps to better understand the underlying pathophysiological mechanisms. 

We studied the interaction between human coronary artery endothelial cells (HCAECs) and *S. agalactiae *by genome wide high density microarrays. The results of this study indicate that GBS are able to regulate transcription of a wide range of genes, involved in infection, inflammation, and apoptosis.

## 2. Materials and Methods

### 2.1. Bacterial Strain

*Streptococcus agalactiae *Lancefield's group B (ATCC®13813 strain Lehmann and Neumann, serotype V, septic, nonhemolytic) was obtained from ATCC® (LGC Promochem, Wesel, Germany). Bacteria were grown overnight on tryptic-soy agar plates supplemented with 5% sheep blood (Institut für Hygiene und Medizinische Mikrobiologie, University of Wuerzburg, Germany) and cultured in 20 ml lysogeny broth (LB) medium, supplemented with 1% trypton, 0,5% yeast extract, and 1% NaCl at 37°C for 90 minutes. A final bacteria concentration of 2 to 4 × 10^9^/ml was achieved after adjustment of the optical density at 600 nm.

### 2.2. Endothelial Cell Culture

Primary human coronary artery endothelial cells (HCAECs, cryopreserved, third passage) were obtained from Lonza (Basel, Switzerland) and cultured in EBM2 medium (Lonza), and supplemented with EGM-2-MV singleQuots (Lonza) with 5% FBS. Endothelial cells were incubated at a humified atmosphere of 5%CO_2_ at 37°C. All cells were subcultured at a 1 : 6 ratio and were used for experiments from passages 5 to 16. After reaching 80% confluence, endothelial cells were washed three times with phosphate buffered saline (PBS) and detached from culture flasks using Trypsin-EDTA solution. The reaction was stopped with TNS solution (Cambrex), and cells were resuspended in complete medium.

### 2.3. HCAEC Infection

GBS, diluted in LB medium, were added in multiplicity of infection (MOI) of 20 to HCAEC. LB medium with HCAEC was used as uninfected controls. 

For gene array analysis, cells were trypsinised, resuspended in trypsin neutralization solution (TNS, Cambrex), and centrifuged at 800 rpm for 5 minutes after 6 hours of infection, because preliminary data have shown that the maximum of gene regulation occurs between 4 and 8 hours. For additional conformation experiments, infection was stopped after 4, 8, and 24 hours. Confirmation of gene array data was selectively carried out by Real-time RT-PCR, western blot analysis, and ELISA at different time points. For Real-time RT-PCR HCAECs were detached from cell culture dishes by a cell scraper, snap-frozen and stored at −80°C until RNA isolation. 

For western blotting, protein extracts were prepared by detaching cells, followed by centrifugation at 800 rpm for 5 minutes. Cell pellets were carefully washed once with 4 ml phosphate buffered saline solution (PBS) and centrifuged again. Cells were resuspended in 100 *μ*l PBS and 100 *μ*l SDS stop solution (200 mM Trizma-Base pH 6.7, 15% Glycerin, 0,03% bromphenole blue, 6% sodium dodecyl sulfate (SDS), ß-mercaptoethanol, 9 : 1, 95°C) for 5 minutes at 95°C. Until further analysis, protein extracts were stored at −20°C. 

In parallel, cell culture supernatants were collected and frozen at −20°C until further analysis by ELISA (uPA). For IL-8 and CXCL-6 ELISA, cells were detached in 150 *μ*l PBS, snap-frozen, and stored at −80°C.

#### 2.3.1. RNA Extraction, cDNA Synthesis, and Array Analysis

For RNA extraction, cell pellets were resuspended in ß-mercaptoethanol and RLT lysis buffer (Qiagen, Hilden, Germany). For disruption and homogenization lysates were loaded on QiaShredder spin columns (Qiagen) and centrifuged at 13.000 g for 2 min. RNA purification was carried out using RNeasy spin columns (Qiagen) according to the protocol of the manufacturer. RNA was eluted in 35 *μ*l RNAse-free water, and RNA concentration was determined photometrically (Nanodrop ND-1000, NanoDrop Technologies, Wilmington, USA). 

For gene array analysis, quality of RNA was determined using a BioAnalyzer (Agilent Technologies, Waldbronn, Germany), and RNA integrity numbers (RINs) were between 9.6 and 9.7. Two *μ*g of total RNA were reverse transcribed, performing second strand synthesis (One-Cycle cDNA synthesis Kit, Affymetrix, Santa Clara, USA). Synthesis of biotinylated cDNA was carried out using the IVT Labelling Kit (Affymetrix). Fifteen *μ*g of the fragmented, labelled cRNA were hybridized to Affymetrix HG-U133 Plus 2.0 arrays. Fluorescence intensity was measured by a GeneChip Scanner 3000 (Affymetrix). For normalization and data analysis, software from the bioconductor project (http://www.bioconductor.org/) was used. After normalization by variance stabilization [15], data quality was verified by density plots, degradation plots, and box plots. Data analysis was based on a more than 3-fold difference compared to unstimulated HCAEC. Resulting genes were functionally clustered according to Gene Ontology using Gene Ontology Tree Machine (GOTM) (http://dbmi.mc.vanderbilt.edu/).

For Real-time RT-PCR, cDNA synthesis was performed using the QuantiTect Reverse Transcription kit (Qiagen) for 25 minutes at 42°C and 3 minutes at 95°C. cDNA was stored at −20°C until further analysis.

### 2.4. Real-Time RT-PCR

Quantitative Real-time RT-PCR for *CXCL6, TLR2, and TLR4* quantification was carried out using the LightCycler instrument. To normalize Real-time PCR data, serially diluted cDNA copies of the housekeeping gene *δ*-aminolevulinic acid synthase (h-ALAS) were coamplified. Analysis was performed comparing the number of cDNA copies of the samples with the h-ALAS copies in each sample, respectively. 45 cycles of repeated denaturation (95°C 9 sec), annealing (54°C 15 sec), and elongation (72°C 25 sec) were performed [16]. The PCR Mastermix (LightCycler Fast Start Master Hybridization, Roche, Mannheim, Germany) contained 0,125 *μ*M of each primer (*CXCL6:* 5′-ttgcacttgtttacgcgtt, 5′-tcagtttttcttgtttccactgt; *TLR2*: 5′-tgtcttgtgaccgcaatggta, 5′-gcttgaaccaggaagacgat; *TLR4*: 5′-ggagccctgcgtggaga, 5′-tatgccccatcttcaattgtc) and 0,15 *μ*M of the hybridization probes (*CXCL6:* 5′-gcaagtttgtctggacccgga-FL, 5′-LC640-gccccttttctaaagaaagtcatccagaa-p; *TLR2*: 5′-ctacagaggtgtgtgaacctccaggc-FL, 5′-LC640-ctggtgctgacatccaatggaattaac-p; *TLR4*: 5′-cccttcaccccgattccattgct-FL, 5′-LC640-cttgctaaatgctgccgttttatcacg-p), labeled with fluorescein and LC Red 640, respectively.

### 2.5. Western Blotting

Cellular proteins were loaded onto SDS-polyacrylamid stacking gels (3%), separated in running gels (12%), and run for 1 hour at 160 V in Tris-SDS-glycine electrophoresis buffer according to Laemmli. Proteins were electrotransferred to polyvinylidene fluoride membranes (Immobilion-P 45 *μ*m, Millipore, Bedford, USA) at 1 A for 60 minutes at 4°C. After being blocked for one hour in Tris Buffered Saline/Tween (25 mM Tris pH7.6, 150 mM NaCl, 0,05% Tween20) containing 6% dry milk, membranes were incubated with primary antibodies (HMOX-1: 1 : 250, milk, mouse, BD Bioscience Pharmingen, San Jose, USA; Bim: 1 : 100, milk, rabbit, BD Bioscience Pharmingen) overnight at 4°C. Membranes were washed briefly and incubated with horse-raddish-peroxidase-conjugated antirabbit or antimouse IgG (goat anti-mouse/rabbit HRP, 1 : 3000, milk, goat, Bio-Rad, Munich, Germany) for one hour at room temperature. Immunoreactivity was detected using an ECL or ECL-plus detection kit (Amersham pharmacia biotech, Freiburg, Germany). The protein bands on X-ray films (Fuji Photo Film GmbH, Düsseldorf, Germany) were scanned, and the intensity of bands was analyzed using NIH Image software (version 1.61). Loading control was performed with ß-actin (1 : 20, milk, rabbit, Santa Cruz Biotechnology Inc., Santa Cruz, USA).

### 2.6. Protein Quantification by ELISA

COX2 (TiterZyme EIA, Assay Designs, Inc., Michigan, USA), IL-8 (Quantikine kit, R&D Systems, Wiesbaden, Germany), and uPA (IMUBIND®, American Diagnostica, Stamford, USA) levels were quantified by ELISA, according to the suppliers protocol. Mean fluorescence was quantified by measurement of optical density, using the SoftMax System (Molecular Devices GmbH, Ismaning, Germany).

### 2.7. Data Analysis

Data represent means +/− standard errors of the means (SEM). Differences between groups were tested using Student's  *t*-test. Differences were considered to be significant at *P* < .05. Confirmation experiments were repeated 3 to 5 times.

## 3. Results

### 3.1. Gene Expression Patterns of HCAEC after Infection with S. agalactiae

In total, 124 genes were differentially expressed (89 upregulated, 35 downregulated) based on a more than 3-fold difference to control HCAEC (see Supplementary Material available online at doi: 10.1155/2011/971502). Signal log ratios (base e) varied between −2.7 and 1.5 comparing control cells versus HCAEC after 6 hours of coincubation with *S. agalactiae.* The highest level of induction was observed for genes encoding transcriptional regulation (14.3-fold upregulated); the highest level of inhibition was achieved in genes involved in DNA-specific binding (4.5-fold downregulated). Raw data are available at http://www.ncbi.nlm.nih.gov/projects/geo/, accession number: GSE15495.

Differencial expression of selected genes (Table 1) was validated by one additional method, including Western blots, Real-time RT PCR, and ELISA assays. We selected regulated genes, which are involved in apoptosis, hemostasis, oxidative stress response, infection, and inflammation for further analysis.

### 3.2. Upregulation of the BCL2-Like-Interacting Protein (Bim) by GBS

Bim belongs to the group of proapoptotic BCL2 members. The initiation of apoptosis after GBS infection is still largely unknown. Previously, we could demonstrate cleavage of Caspases 3 and 8 after infection of HCAEC with *S. agalactiae* (data unpublished).

**Figure 1:**
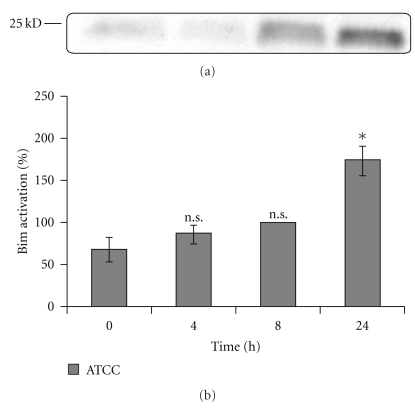
Time course of Bim upregulation in HCAEC induced by GBS. (a) Autoradiograph of western blot analysis of cell culture lysates demonstrating upregulation of Bim after 4, 8, and 24 h in comparison to unstimulated HCAEC. (b) Quantitative analysis of the Bim signaling pathway. Data shown represent mean expression in percent ± the standard error of the mean (SEM) of up to 4 independent experiments. The results after 8 hours are defined as 100 percent. ∗ Indicates statistical significant difference (*P* < .05); n.s.: no significant difference.

Expression of Bim was demonstrated by Western blot analysis of cell culture lysates. Bim protein levels were increased by 1.3-fold after 4 hours, 1.5-fold after 8 hours, and 2.6-fold after 24 hours of infection, compared to uninfected cells (Figure 1).

### 3.3. Upregulated Expression of the Heme-Oxygenase-1 (HMOX-1) by GBS

Heme oxygenase 1 plays an important role in the regulation of apoptosis and cell protection during inflammatory processes. Expression of HMOX-1 is species- and cell-specific, induced by oxidative stress, due to LPS, ischemia, or cytokines [17]. 

**Figure 2:**
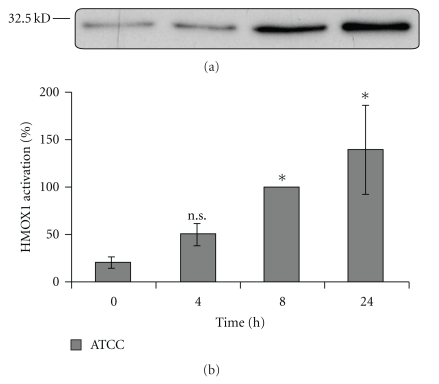
Time course of HMOX-1 upregulation in HCAEC induced by GBS. (a) Autoradiograph of western blot analysis of cell culture lysates demonstrating upregulation of HMOX-1 after 4, 8, and 24 h in comparison to unstimulated HCAEC. (b) Quantitative analysis of the HMOX-1 signaling pathway. Data shown represent mean expression in percent ± SEM of up to 4 independent experiments. The results after 8 hours are defined as 100 percent. ∗ Indicates statistical significant difference (*P* < .05); n.s.: no significant difference.

Infection of HCAEC with the septicemic *S. agalactiae *strain ATCC® 13813 led to HMOX-1 upregulation, compared to unstimulated cells (2.3-fold after 4 hours, 4.7-fold after 8 hours, and 6.6-fold after 24 hours of infection, Figure 2).

### 3.4. Downregulation of CXCL6 by Real-Time RT PCR

CXCL6 is a strong mediator of inflammation, attracting mainly neutrophils [18], but also eosinophils, lymphocytes, and monocytes [19]. CXCL6 production can be induced by IL-1-ß, TNF-alpha, hypoxemia, and LPS [20].

**Figure 3:**
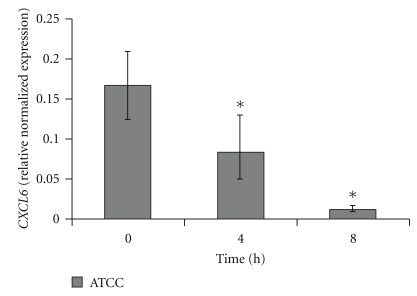
Gene expression of *CXCL6* was analyzed by quantitative Real-time RT-PCR. Data shown represent normalized mean expression of *CXCL6* ± SEM of up to 4 independent experiments. *CXCL6* expression was normalized against h-ALAS housekeeping-gene. ∗ Indicates statistical significant difference (*P* < .05).

Downregulation of *CXCL6 *after GBS stimulation was observed (2-fold after 4 hours and 12.9-fold after 8 hours of infection). After 24 hours of infection, no CXCL6-RNA was detectable (Figure 3).

### 3.5. Downregulation of TLR2 and TLR4 by Real-Time RT PCR

TLR 2 and TLR4 are major receptors of the human innate immune response against various pathogens, interacting with lipoteichoic acid and bacterial LPS, respectively [21].

**Figure 4:**
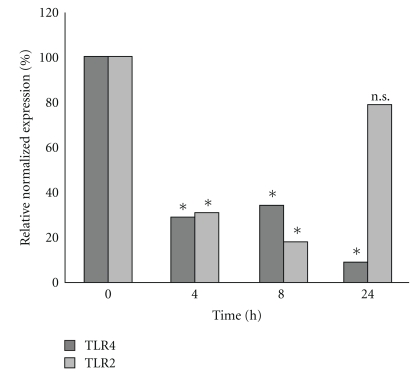
Gene expression of *TLR2 *and* TLR4* was analyzed by quantitative Real-time RT-PCR. Data shown represent mean expression of *TLR2 *and* TLR4* in percent. *TLR* expression was normalized against h-ALAS housekeeping-gene. ∗ Indicates statistical significant difference (*P* ≤ .05).

TLR4 expression was reduced after GBS stimulation (3.4-fold after 4 hours, 2.9-fold after 8 hours, and 11-fold after 24 hours of infection). We also observed downregulation of *TLR2 *(4.3-fold after 4 hours, 3.8-fold after 8 hours, and 1.2-fold after 24 hours of infection, Figure 4).

### 3.6. Downregulation of Interleukin 8 (IL-8) in Cell Culture Lysates

Interleukin 8, a member of the CXC chemokine superfamily, attracts neutrophils and monocytes to sites of inflammation. Production of IL-8 can be induced by different cytokines, viral infections, or gram negative bacteria [22].

IL-8 secretion was reduced by factor of 2.5 (4 h), 1.7 (8 h), and 1.5 (24 h).

### 3.7. Upregulation of Urokinase (uPA) in Cell Culture Supernatants

**Figure 5:**
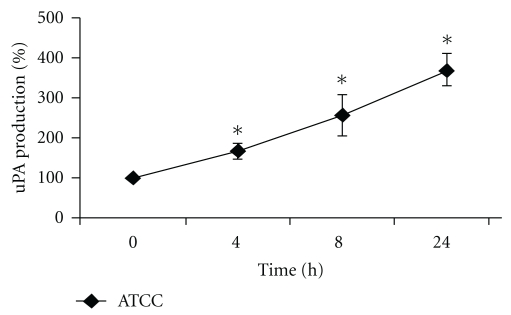
Time course of uPA production by HCAEC coincubated with GBS. uPA released from HCAEC was determined in the cell culture supernatants by ELISA as described in section 2. Data shown represent mean production in percent ± SEM of 3 independent experiments. The unstimulated control is defined as 100 percent. ∗ Indicates statistical significant difference (*P* < .05).

Urokinase regulates fibrinolysis by plasminogen activation. Therefore, hemorrhagic dysregulation, observed in GBS infected neonates might be enhanced from the observed upregulation of urokinase. We found upregulation in urokinase levels in HCAEC cell culture supernatants (1.6-fold after 4 hours, 2.6-fold after 8 hours, and 3.7-fold after 24 hours of infection, Figure 5).

### 3.8. Upregulation of Cyclooxygenase-2 (COX2) in Cell Culture Lysates

COX2 is induced during inflammation among others in macrophages, monocytes as well as in endothelial cells [23]. No data are known about COX2 induction in HCAEC after GBS infection.

We observed a weak upregulation of COX2 expression after 8 hours of infection (1.5-fold), whereas COX2 levels remained unchanged after 4 and 24 hours of infection compared to uninfected HCAEC.

## 4. Discussion

The present study demonstrates the use of cDNA microarray technology to characterize transcriptional responses in HCAEC coincubated with GBS.

The major new findings of this study are as follows. (i) GBS is capable to differentially regulate genes in HCAEC. (ii) GBS leads to upregulation of the proapoptotic BCL2-like-interacting protein (Bim) and of heme-oxygenase-1 (HMOX-1). (iii) GBS causes downregulation of the chemokines *CXCL6* and IL-8. (iv) Urokinase (uPA) and cyclooxygenase-2 (COX2) were activated after GBS infection.

Doran et al. [24] performed a study analyzing the interaction between *S. agalactiae *and human brain microvascular endothelial cells. In contrast to our study, this group used a haemolytic strain and revealed ß-hemolysin/cytolysin as the most important pathophysiological player in a murine model of hematogenous meningitis. 

Our data show that the proapoptotic protein Bim is upregulated during the course of GBS infection. One reason for Bim upregulation during infection with *S. agalactiae *could be elevated levels of cytokines and chemokines in HCAEC [25]. Bim might cause the release of mitochondrial cytochrome c, activating the intrinsic way of apoptosis induction [25]. Our own previous results (data unpublished) indicate that GBS can cause a breakdown of the mitochondrial membrane potential in HCAEC, reflecting an initiation of programmed cell death. 

Activation of Toll-like receptors leads to an augmented phosphorylation and inactivation of Bim, which in consequence avoids apoptosis [26]. Our group was able to demonstrate a downregulation of *TLR2* and *TLR4* after infection with the septicemic GBS strain ATCC® 13813 after 4 h, 8 h, and 24 h, respectively, compared to unstimulated cells. GBS-induced downregulation of TLR4 might be responsible for Bim-induced apoptosis, which might support tissue invasion of GBS. 

According to Smith et al. [27], who observed an upregulation of HMOX-1 after stimulation with *Streptococcus pneumoniae*, we could demonstrate activation of HMOX-1 in HCAEC coincubated with *Streptococcus agalactiae. *After activation, HMOX-1 cleaves hemoglobin into carbon monoxide (CO), iron (Fe^2+^), and biliverdin, followed by conversion into bilirubin [28]. All degradation products of heme can potentially be both toxic and protective. Carbonmonoxide leads to vasodilatation and lower inflammatory response [29]. Furthermore, CO impairs platelet aggregation. 

Our own group could previously demonstrate that GBS is able to induce platelet aggregation [30]. In consequence, upregulated expression of HMOX-1 could reflect a protective mechanism against thrombotic complications after infection with GBS. 

In addition, overexpression of HMOX-1 might lead to elevated bilirubin levels [30], potentially interfering the change from fetal to adult hemoglobin [31]. Hyperbilirubinemia might be responsible for neurological long-term sequelae, observed after GBS newborn infections [32]. Furthermore HMOX-1 could indirectly and directly inhibit apoptosis by minimizing reactive oxygen species (ROS) [33] and by regulation of NF*κ*B signaling [34]. In summary, induction of HMOX-1 by GBS could represent a response of HCAEC to GBS-induced pathomechanisms such as apoptosis and inflammation.

*CXCL6*, which encodes a strong chemotactic protein mainly for neutrophils, was markedly downregulated in HCAEC after infection with *S. agalactiae*. Impaired *CXCL6* levels could be responsible for lower neutrophil recruitment and activation during GBS sepsis. In addition, CD4 positive T-cells, activated by CXCL6, secrete augmented levels of IgG, leading to opsonisation of microorganisms and thereby to facilitated phagocytosis [35]. In parallel to *CXCL6*, a further chemokine, IL-8, was downregulated after GBS infection, potentially leading to impaired chemotaxis of monocytes and neutrophiles. Futhermore, our own data revealed that production of a third chemotactic protein, monocyte chemoattractant protein (MCP)-1, was downregulated after incubation with *S. agalactiae *unpublished. Taken together, these results indicate either a mechanism of the host to avoid excessive inflammation or a bacterial strategy to suppress innate immune response.

The serine protease urokinase plays an important role in fibrinolysis by activating plasminogen to plasmin. Urokinase-type plasminogen activator upregulation might explain the simultaneously observed hemorrhagic and thrombotic complications during GBS sepsis [30]. Cocultivation of HCAEC with *S. agalactiae *revealed marked upregulation of urokinase, as determined by ELISA assays. Elevated urokinase levels could lead to resolution of platelet-bacteria-thrombi resulting in facilitated tissue invasion of GBS. 

In parallel to urokinase, we found a second thrombolytic factor, cyclooxygenase 2, to be upregulated during GBS infection, whereas COX1 is known to be constantly expressed [36]. Imbalance of prostacyclin (produced by COX2) and thromboxane A2 (produced by COX1) might be responsible for hemorrhagical complications [37]. Prostacyclin-induced vasodilatation might facilitate spread of bacteria in the blood vessel system. Interestingly, COX2 levels were only weakly increased after 4 hours of infection. Infection with a highly septicemic bacteria strain leads to marked increase of cyclooxygenase 2 expression [38]. However, in this study the authors used LPS and macrophages.

In summary, our data demonstrates that *S. agalactiae* might be able to escape innate immune mechanisms and to induce hemorrhagical complications in HCAEC. Induction of apoptosis, bleeding, and vasodilatation could explain the invasive growth during GBS sepsis. 

Knowledge of molecular mechanisms during GBS infection will contribute to a broader understanding of the interaction of streptococci with the endothelium and, in consequence, will support the development of new therapeutical strategies against this severe complication.

## Figures and Tables

**Table 1:** Differential expression of selected genes Confirmation of gene expression profiles of the 6 listed selected genes was performed by Real-time RT PCR, western blot or ELISA assays.

Downregulated genes	
Gene	Regulation [fold]

Interleukin 8	3,9
Chemokine (C-X-C motif) ligand 6 (granulocyte chemotactic protein 2)	3,9

Upregulated genes	

Gene	Regulation [fold]

Heme oxygenase (decycling) 1	10,7
Prostaglandin-endoperoxide synthase 2 (prostaglandin G/H synthase and cyclooxygenase)	9,9
BCL2-like 11 (apoptosis facilitator)	6,0
Plasminogen activator, urokinase /// plasminogen activator, urokinase	3,4

## Abbreviations

ATCC: American type culture collection
BIM: BCL2 interacting protein
COX2: Cyclooxygenase 2
CXCL6: Chemokine (CXC motif) ligand 6
GBS: Group B streptococcus
HCAEC: Human coronary artery endothelial cells
IL8: Granulocyte chemotactic protein 1
LB: Lysogeny broth
TLR: Toll-like receptor
uPA: Urokinase-type plasminogen activator.
